# The Art of Medicine: Applying the Visual Thinking Strategy to Radiology

**DOI:** 10.7759/cureus.67745

**Published:** 2024-08-25

**Authors:** Madison Wulfeck, Jeffrey Waltz, Jordan H Chamberlin, Jeanne G Hill

**Affiliations:** 1 Department of Radiology, Medical University of South Carolina, Charleston, USA

**Keywords:** learning curriculum, medical imaging, perceptual errors, radiology, visual thinking strategy

## Abstract

Objective: The purpose of this project was to develop a formal visual arts training curriculum and evaluate if there was improvement in the observational and descriptive skills of first- and second-year medical students for radiologic images.

Materials and methods: A demographic survey and an initial pre-test of 12 radiologic images were administered asking an open-ended question to describe the image and to identify the abnormality in their own words. Three virtual one-hour sessions of visual thinking strategy (VTS) training occurred, and an immediate post-test and a six-month post-test were administered, each with images different from the pre-test, as well as a final questionnaire. All tests were independently graded by two graders with a previously established grading rubric. Differences in scores were analyzed using paired T-tests.

Results: Thirty-nine medical students participated. The mean pre-test score was 62.2 +/- 18.6, and the mean post-test score improved by 41.7 +/- 17.9 points (p<0.01) to an average score of 103.9 +/- 20.4. Nine participants were lost to follow-up at six months, and the average six-month post-test score was 110.2 +/- 29.1 for a mean improvement of 9.3 +/- 13.1 points (p=0.320) from the initial post-test.

Conclusion: There was a significant improvement in observational and descriptive skills in first- and second-year medical students when describing radiologic images, which was retained after six months. A formal VTS curriculum could play a beneficial role in medical student and radiology training programs not only to improve observational skills but also to address perceptual errors in diagnostic imaging.

## Introduction

Interpretation of medical imaging is a complex cognitive process that requires identification, summation, and efficient communication, which is subject to variability at each juncture. It has been well-established that many errors in radiology are perceptual in nature and not a result of interpretation errors [1,2]. To put it plainly, if you cannot see something, you will not be able to interpret and apply that interpretation to guide the appropriate management of the patient. There are many reasons why perceptual errors predominate, including reader fatigue, distractions such as phone calls, increased speed of interpretation, and the phenomenon known as “satisfaction of search” [3].

In a visual search of a diagnostic image, the eyes must systematically search by concentrating the central visual field over different regions of interest. The complexity of this task is further compounded by the review of hundreds, if not thousands, of images, comprising a single cross-sectional examination. Eye gaze tracking technology used to track eye movements and visual dwell times is able to quantify the amount of time the viewer’s visual gaze is spent on certain areas of the image, which has shown a direct correlation between dwell time in a region and abnormality detection in that same region. Most importantly, it has also been shown that missed findings are frequently located in these regions of higher visual dwell time [3]. Interestingly, in a previous study by Mallett et al. (2014), visual dwell time is longer and frequently returns to the region of the image containing a finding that is ultimately missed [4]. These findings beg the question of whether the radiologist is actually looking at the abnormality but fails to perceive it, what kind of training will enhance his/her ability to recognize the abnormality consciously?

Visual literacy is an acquired skill that enables an individual to approach, inventory, and objectively analyze an image or object prior to making interpretations or drawing conclusions [5]. In the visual arts field, this expertise is used at every level in order to make explicit what an artist is trying to evoke in an image. Visual Thinking Strategy (VTS) is a methodology that uses visual art to improve critical thinking and communication skills by encouraging students to observe and analyze images through open-ended questions. Systematic and objective instruction of this skill may prove advantageous for radiologists who rely heavily on the identification of visual abnormalities and are prone to perception error.

The purpose of this project is to evaluate the effect of formal visual arts training on the observational and descriptive skills of first- and second-year medical students as they apply to radiologic images. The goal was to use visual literacy to bridge the gap between subconscious recognition of an image abnormality and its explicit description and interpretation, which has the potential to address radiologists’ perceptual errors. Because of the paucity of implementation of formal art training in medical education, we hope to demonstrate the objective utility of increasing the visual literacy of medical students early in training and to develop a valid radiology-targeted visual literacy curriculum that may potentially be incorporated into medical education and residencies across institutions in the future.

## Materials and methods

This study received approval by the Medical University of South Carolina (MUSC) Institutional Review Board (No. Pro00086970). This article was previously posted to the Research Square preprint server on October 14, 2023. 

Recruitment and pre-test

First- and second-year medical students were recruited to volunteer to participate in the study via class-wide email and in-class announcements. Consent was understood and implied by the medical students’ participation in the research study. There was no target sample size as the study was limited by the volunteer participation of the medical students. Medical students were excluded from the final analysis if they had not completed all three of the VTS sessions or did not complete one of the three tests. In an initial demographic survey, year of medical school, gender, and prior visual arts training were collected. Students then completed a pre-test consisting of 12 radiologic images that were previously evaluated and objectively characterized by two radiology residents and an attending radiologist. Each test consisted of nine plain films and three computed tomography (CT) single images. The participants were given the following directions: Please look at each image carefully. Describe any and all aspects of the image underneath the image using as much description as necessary. Then, identify the abnormality (in words) and describe it in detail. Please note that you are not expected to make a diagnosis or know the exact anatomy. You may also resize the image and use additional pages for your description.

**Figure 1:**
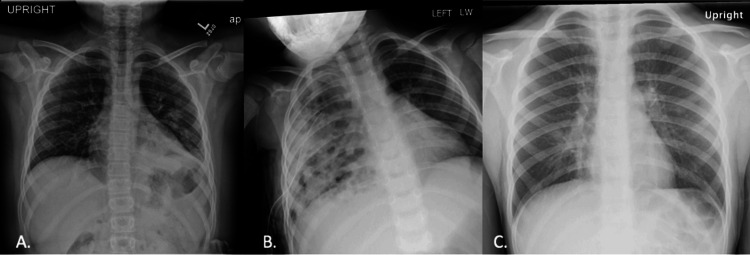
Pediatric chest radiographs A. Pediatric chest radiograph of left lower lobe pneumonia on the pre-test with patchy airspace opacities in the left lower lobe with silhouetting of the left hemidiaphragm. B. Right lung cystic/necrotizing pneumonia on the post-test with heterogeneous patchy airspace opacities throughout the right lung and a right-sided thoracostomy tube present. C. Interstitial/viral pneumonia on the six-month follow-up post-test with central predominant interstitial opacities. All images were acquired from the internal institutional Picture Archiving and Communication System (PACS).

Each test had similar images between the tests but with different pathologies. For example, question number five on each of the tests was a pediatric chest radiograph with a different morphology of pneumonia (Figures 1A-1C). The intent was to have the student utilize similar visual search patterns and descriptions without using the same images. The pre-tests were completed within one week prior to the VTS training intervention.

Intervention: VTS training

Three one-hour sessions of visual thinking strategy (VTS) training occurred virtually utilizing WebEx (webex.com, San Jose, CA) between September and October 2020 for a total of three hours. Given the time constraints and scheduling difficulties of medical school, recordings were provided to all of the participants after the sessions. The program was developed by the Gibbes Museum of Art in conjunction with the principal investigators, and sessions were taught by the museum curator and trained volunteer art educators. Images and exercises for each of the VTS sessions were chosen by museum curators at the Gibbes Museum of Art. Standard questions sought to be answered during VTS include: 1. What’s going on in this image? 2. What do you see that makes you say that? 3. What more can we find?

**Figure 2:**
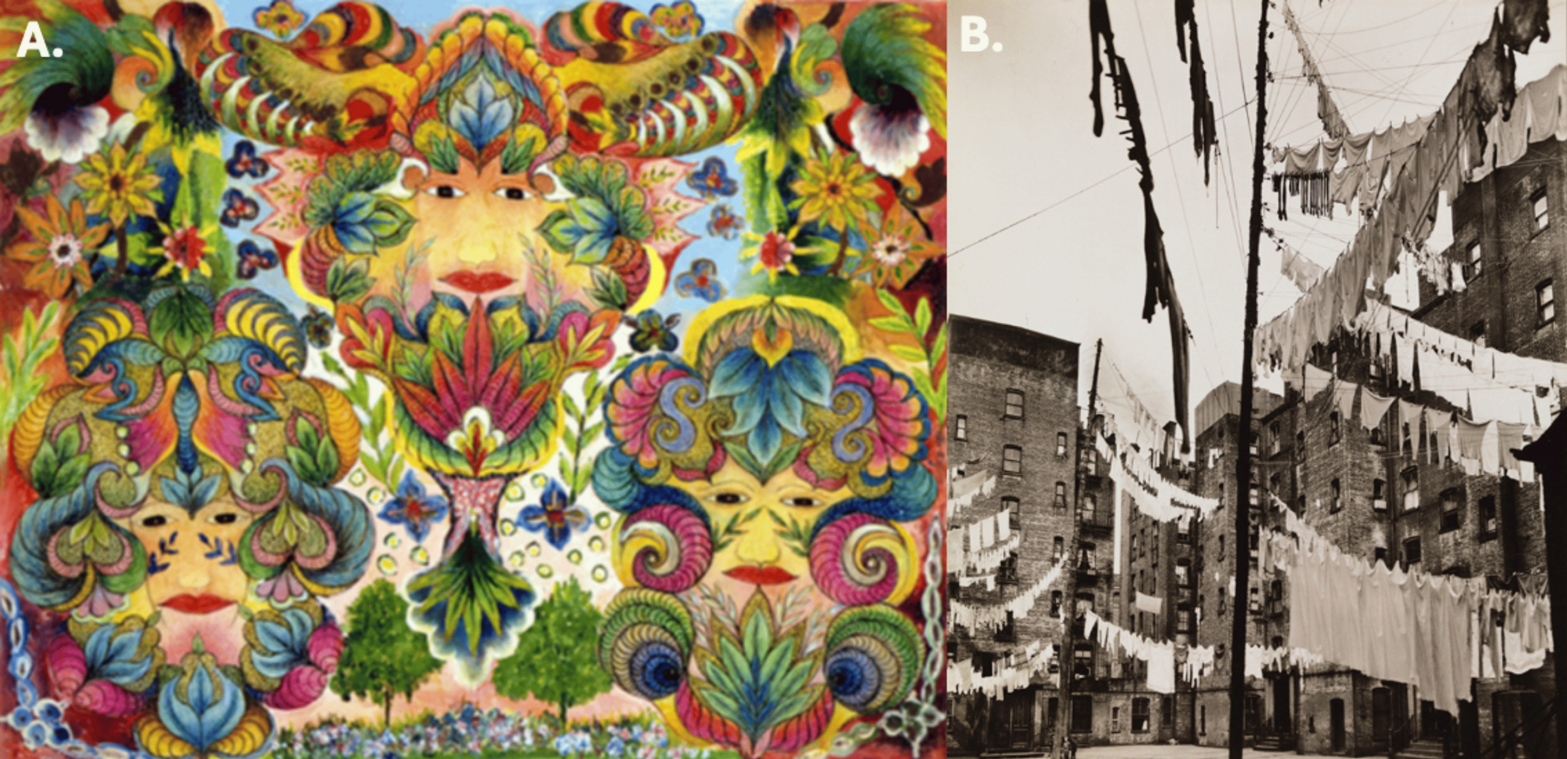
Artwork discussed during the first VTS session (A) Painting entitled “Designs, Wrightsville Beach, 1968” by Minnie Evans, which was displayed at the Gibbes Museum of Art. Image retrieved from: https://www.gibbesmuseum.org/news/breaking-down-barriers-300-years-of-women-in-art/designs-web/ (B) Photograph entitled “Washlines from the series Changing New York, 1936” by Berenice Abbott which was also displayed at the Gibbes Museum of Art. Image retrieved from: https://www.mcny.org/story/fine-line-art-clothesline. VTS: Visual thinking strategy

The first session consisted of an introduction to VTS and a brief demonstration by the art educators. The students were organized into three small breakout rooms where they discussed a painting (Designs, Wrightsville Beach, 1968 by Minnie Evans, Gibbes Museum of Art) (Figure 2A) and a photograph (Washlines from the series Changing New York, 1936 by Berenice Abbott, Gibbes Museum of Art) (Figure 2B) from the museum’s collection using VTS. This painting was chosen because of the colors that were incorporated as well as the patterned approach to the composition. The photograph was chosen because of its black-and-white nature to encourage deeper reflection about the setting. Each group’s findings were then presented to the whole group. The goal of the first session was to implement the VTS methodology to a patterned color painting as well as a black and white photo.

**Figure 3:**
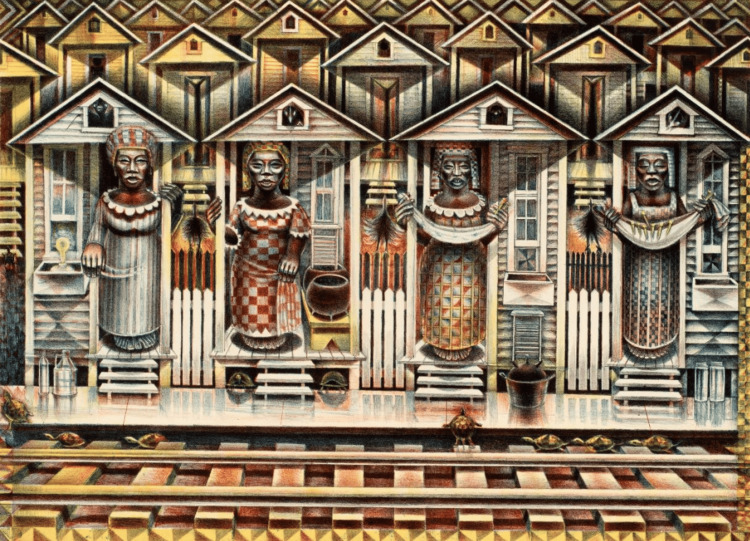
Artwork discussed during the second VTS session Four Seasons, 1990 by John Biggers, Gibbes Museum of Art. Image retrieved from: https://apfineart.com/artworks/categories/1/55-john-biggers-four-seasons-1990/ VTS: Visual thinking strategy

The second session included an analysis of a lithograph (Four Seasons, 1990 by John Biggers, Gibbes Museum of Art) (Figure 3) through discussion and a writing exercise where students were tasked with creating a poem from the point of view of a figure in the work of art. Each group then shared a poem with the whole group. This lithograph was chosen because of the deeper meanings that could be derived from the figures as well as the subtle differences between the figures.

**Figure 4:**
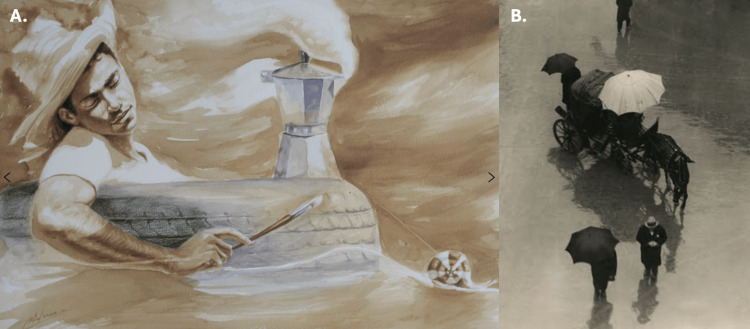
Artwork discussed during the third VTS session (A) “SOS for a Shipwreck at Sea, 2023” by Reynier Llane (Cuban-American, b. 1985). Coffee and acrylic on paper. Image retrieved from: https://www.gibbesmuseum.org/exhibitions/reynier-llanes-passages/135 (B) “The Happy Family – The Poor Relative, 1955” by Martin Munkacsi, Gibbes Museum of Art. Image retrieved from: https://ashliebutler.wordpress.com/wp-content/uploads/2014/03/brolly-reffed.jpg VTS: Visual thinking strategy

The third and final session consisted of an analysis of a painting (SOS, 2021 by Reynier Llanes, courtesy of Jonathan Green and Richard Weedman) (Figure 4A) and a black-and-white photograph (The Happy Family - The Poor Relative, 1955, by Martin Munkacsi, Gibbes Museum of Art) (Figure 4B) through the creation of a cinquan poem. A Cinquan poem template was provided with the students to fill in blanks that consisted of emotions or thoughts that they experienced while viewing the images. This was meant to provide the student with a way to elucidate their perspectives. Each student created their own poem, which was shared with the breakout groups as well as the larger group. The painting was chosen because of the unconventional medium of coffee, and the photograph was chosen to get the students to describe the emotions of the included figures.

Testing scoring

Scoring for the pre-test and subsequent post- and six-month follow-up tests was based on an objective score out of 9 for pre-determined descriptions that were established by the scorers. For example, Figure 1A grading rubric included one point for each of the following that was described: type of examination, description of views, type of patient, body part imaged, recognition of abnormality in the left lower lobe, elevated left hemidiaphragm, any description regarding the cardiomediastinal silhouette, presence of air bronchograms, and mildly prominent interstitial linear opacities. An additional 3 points were allocated for the overall quality of the description which was multiplied by two. The maximum score for each question was 15 points, which equated to 180 points for the entire test (Table 1).

**Table 1:** Sample grading rubric for one of the pretest questions shown in Figure 1A

Pretest Grading Rubric: Pediatric chest radiograph of left lower lobe pneumonia	
1 point for each of the following:	Type of examination: radiograph
	Description of views: frontal/AP
	Type of patient: pediatric (open physes)
	Body part imaged (chest)
	Recognition of an abnormality in the left lower lung area
	Elevated left hemidiaphragm secondary to volume loss
	Description cardiac silhouette with asymmetric white-ness and/or presence of silhouette sign or not being able to delineate the left hemidiaphragm
	Presence of linear lucencies (air bronchograms)
	Mildly prominent interstitial lines/opacities
Plus 3 points for overall description (0=poor, 1=fair, 2=good, 3=excellent) x 2 for a total of 15 points	

Grading rubrics were established by two radiology residents (one third-year and one fourth-year resident) with the oversight of an attending radiologist. The completed tests were coded for anonymity and all of the tests were graded against the established rubrics at the same time by the same two radiology residents a week after completion of the six-month post-test.

Post-test and six-month follow-up

Post-tests were administered within one week after the completion of the curriculum in addition to a six-month post-test six months after the intervention (Tables 2, 3). Finally, a post-test questionnaire was also administered (Appendix 1). Participants were awarded a $25 gift card upon completion of the post-test and a free one-year membership to the Gibbes Art Museum upon completion of six months of post-test and questionnaire.

**Table 2:** Sample grading rubric for one of the post-test questions shown in Figure 1B

Post-test Grading Rubric: Pediatric chest radiograph of cystic/necrotizing pneumonia	
1 point for each of the following:	Type of examination: radiograph
	Description of views: frontal/AP
	Type of patient: pediatric (open physes)
	Body part imaged (chest)
	Patchy white right lung
	Description of heterogeneity
	Mentioning of some gas within the lung
	Right-sided thoracostomy tube present
	Mentioning the inability to see the right heart border
Plus 3 points for overall description (0=poor, 1=fair, 2=good, 3=excellent) x 2 for a total of 15 points	

**Table 3:** Sample grading rubric for one of the six-month post-test questions shown in Figure 1C

Six-Month Post-test Grading Rubric: Pediatric chest radiograph of interstitial/viral pneumonia	
1 point for each of the following:	Type of examination: radiograph
	Description of views: frontal/AP
	Type of patient: pediatric (open physes)
	Body part imaged (chest)
	Linear opacities
	Greatest around the mediastinum/perihilar regions
	Diffuse pattern
	Slightly hazy everywhere
	Any other incidentals correctly noted/described
Plus 3 points for overall description (0=poor, 1=fair, 2=good, 3=excellent) x 2 for a total of 15 points	

Statistical analysis

Pre-study and post-study scores were aggregated across two readers, reported using means and standard deviations, and interrater correlations were calculated. Standard demographic information and relevant experience were reported using counts and percentages. The primary statistical outcome was the difference in pre- and post-intervention scores as measured by two-way T-tests for paired samples. A standard alpha of 0.05 was used for this study. Correlation between scoring variables, demographics, and relevant experience was given using Pearson’s correlation coefficient. All statistical analysis was performed in R statistical programming v 3.6.2 (R Foundation for Statistical Computing, Vienna, Austria).

## Results

Test data analysis

Out of approximately 162 enrolled first-year medical students and 171 enrolled second-year medical students, a total of 39 first- and second-year medical students (72% female) participated (Table 4). Out of the total, 36% (14) of students had prior programming experience and 10% (four) of students had previous visual arts training. 

**Table 4:** Demographics of participants in the study

Demographics (N = 39)	Mean (SD)/ Count (%)
Age	25.4 (2.9)
Sex	
Male	11 (28.2)
Female	28 (71.8)
Year in medical school	
First year	22 (56.4)
Second Year	17 (43.6)
Any programming experience	14 (35.9)
Any visual arts training	4 (10.3)
Skills in plain films	
(Least confident) 1	12 (30.7)
2	15 (38.5)
3	8 (20.5)
(Most confident) 4	4 (10.3)
Skills in computed tomography	
(Least confident) 1	16 (41.0)
2	12 (30.8)
3	8 (20.5)
(Most confident) 4	3 (7.7)

**Figure 5:**
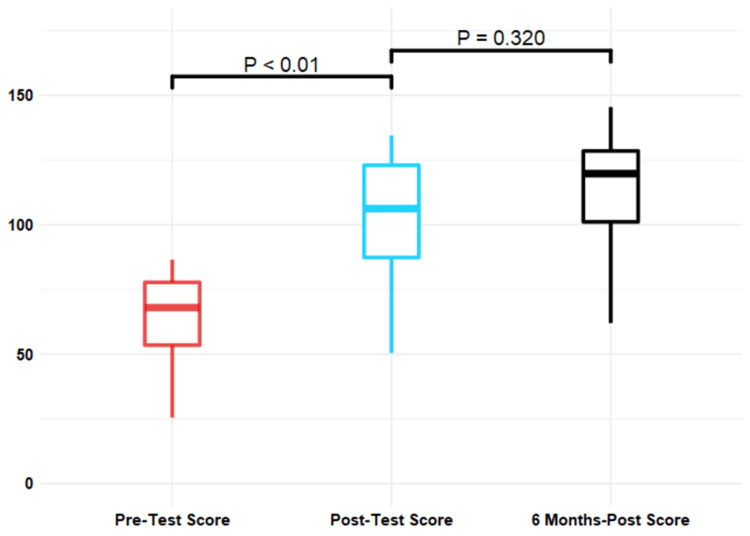
Comparison of scores at three points Post-intervention test scores were significantly higher than pre-intervention test scores. There were no differences in post-intervention test scores at 0 or six months (with nine participants lost to follow-up). P-values were calculated using two-way T-tests for paired samples.

**Figure 6:**
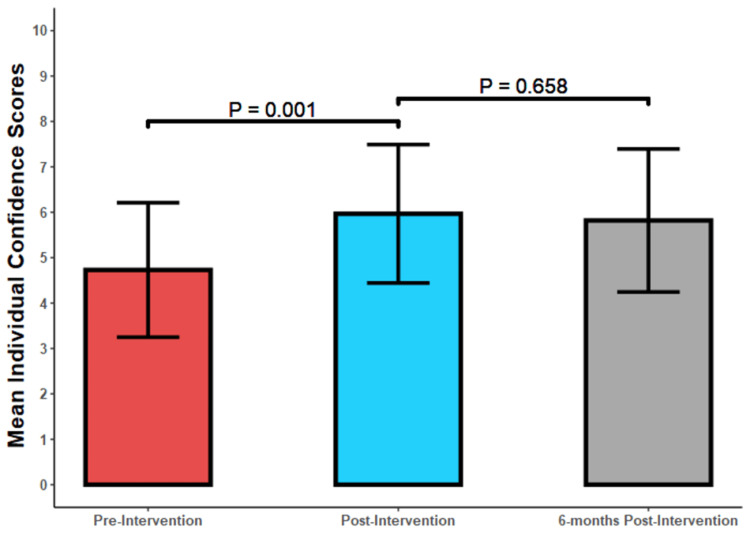
Mean individual confidence scores for the pre-tests, post-tests, and six-month post-tests

The mean pre-test score was 63.3 +/- 19.3 and the mean post-test score improved by 41.9 +/- 16.1 points (p<0.001) to an average score of 105.2 +/- 20.5 (Table 5 and Figure 5). There were nine participants lost to follow-up at six months, and the average six-month post-test score was 110.2 +/- 29.1 for a mean improvement of 5 +/- 27 points (p=0.320) from the initial post-test. The overall difference in score from the beginning of the study to the end of the six-month follow-up was 46.9 (p < 0.001). There was an excellent inter-rater correlation with an inter-rater correlation (ICC) of 0.993. There was a statistically significant increase in the medical students’ mean confidence scores between the pre-test and the post-test (p=0.001) and no statistically significant difference in the mean confidence scores between the post-test and the six-month post-test (p=0.658) (Figure 6).

**Table 5:** Scores at baseline and between intervention and follow-up Scores are reported as averages of raters with mean and standard deviations.  P-values were calculated using two-way T-tests for paired samples. Δ = change in test score. *Nine participants (23.1%) were lost to follow-up. The total number of participants with six-month follow-up tests was 30.

Measure (N = 30)	Mean + SD	P value	t*
Pre-Score	63.3 (19.3)	---	
Post-Score	105.2 (20.5)	---	
6 months-score*	110.2 (29.1)	---	
Pre-Post Δ	41.9 (16.1)	<0.001	-3.45
Post-6 months Δ*	5 (27)	0.320	0.445
Pre-6 months Δ*	46.9 (21.4)	<0.001	-3.16

**Figure 7:**
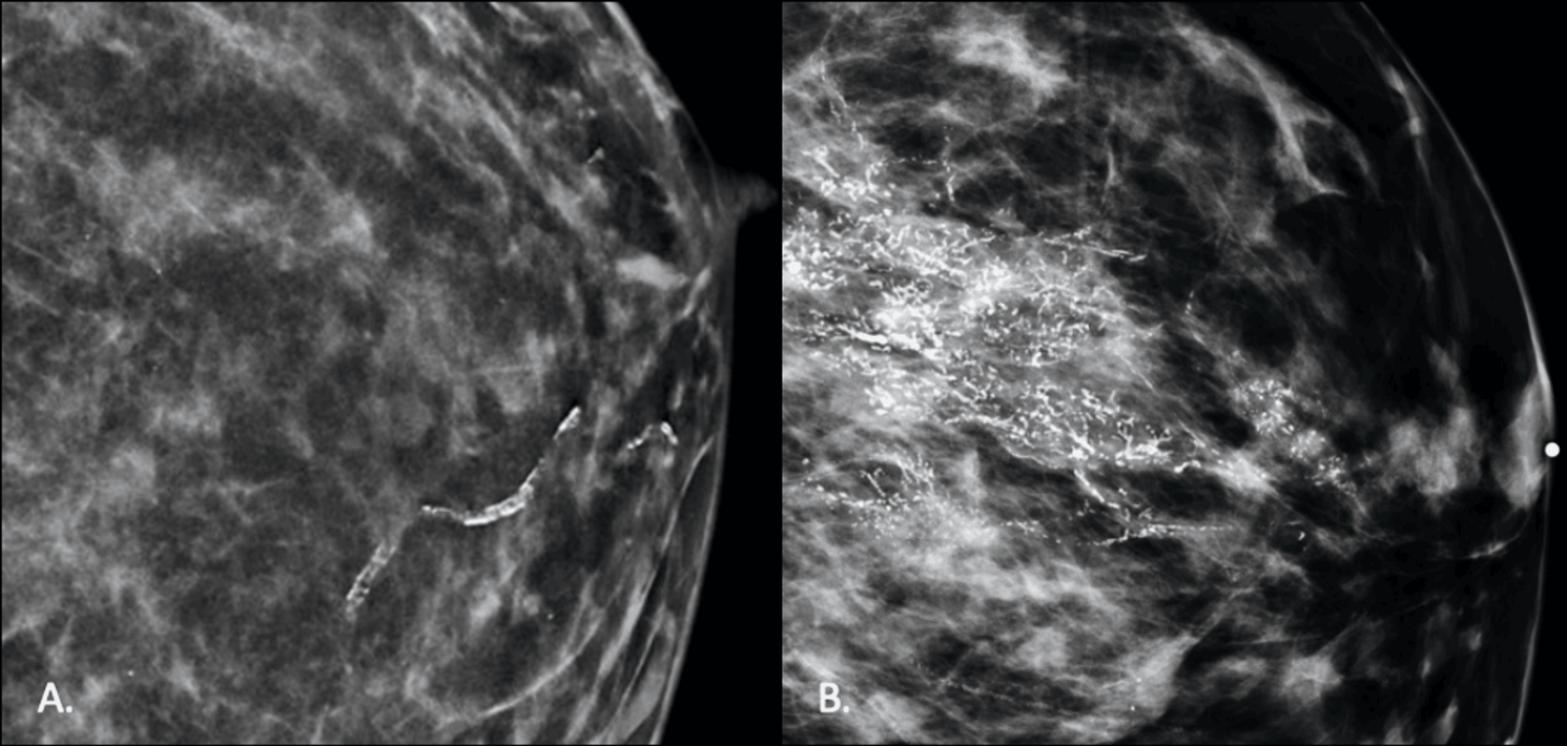
Pre- and post-testing questions using different mammograms (A) Pre-test question of a mammogram demonstrating linear calcifications consistent with benign, vascular calcifications. (B) Post-test question of a mammogram demonstrating tiny, irregular calcifications in a segmental or regional distribution, subsequently shown to be biopsy-proven ductal carcinoma in situ. Images were acquired from the internal institutional Picture Archiving and Communication System (PACS).

Post-test image descriptions were significantly longer and more descriptive. For example, below are the responses to the pre-test question depicting an image of a mammogram with vascular calcifications (Figure 7A) as well as to the post-test question depicting an image of a mammogram with regional pleomorphic calcifications (Figure 7B).

Pre-test response: This is a mammogram of a breast. The breast tissue appears relatively dense (scattered density/lighter patches). It looks like that the abnormality is the calcified duct (very bright line).

Post-test response: This appears to be a mammogram of a breast. You can tell this by the nipple marker at the right side of the image and the outline of the breast here. There is dense breast tissue scattered throughout (lighter whisps). There are also very bright calcifications speckled throughout the center of the breast tissue. These calcifications are much brighter than the surrounding tissue and have a different consistency. I believe this is the primary abnormality. 

**Figure 8:**
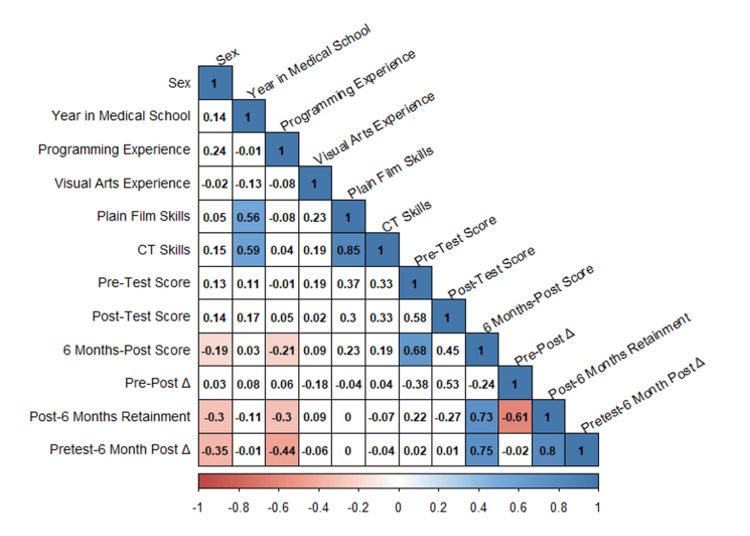
Correlation matrix of demographics and outcomes variables Significant correlations are highlighted in color; nonsignificant correlations are blank.

A correlation matrix of demographics and outcomes variables demonstrated that years in medical school significantly correlated with reported confidence in plain film and CT skills and the pre-test score was significantly associated with the six-month post-test score (Figure 8). Male sex was negatively correlated with six-month post-test score, six-month retainment, and overall pretest to six-month post-test improvement. Prior visual arts experience was included in the initial demographics in an attempt to control for any skill in art analysis that may give the participant an advantage. Prior visual arts experience did not have any significant correlation, positive or negative, with six-month post-test score, six-month retainment, and overall pretest to six-month post-test improvement.

Post-test questionnaire analysis

Two major themes emerged from the post-test questionnaires: the uniqueness of intentional analysis and the validity of open-ended questions as well as collaboration. Many students observed the differences in their search patterns and the pace at which they analyzed the images. Instead of just focusing on one thing, the students were encouraged to identify and analyze as many things as possible, in addition to re-analyzing previous findings when new ideas were introduced. One student shared the following: "I think after the course, I began looking at images more holistically and objectively, not as a medical student or someone who has studied anatomy. Instead of immediately jumping to recognize and identify the anatomy I have been taught and then guessing what aspects of the image did not fit my perception of “normal,” I began to look at the image as a whole, describing it as someone who knew nothing about anatomy, which allowed me to focus on aspects of the image I may have glossed over before."

The students also seemed to appreciate being able to answer many open-ended questions. They noticed that it encouraged significant discussion as well as opened additional avenues for more interesting observations. The most commonly cited criticisms included the overall time commitment of the VTS sessions, the limited social environment of WebEx, and the lack of explanation as to how the strategy can be specifically applied to radiology images.

## Discussion

Our formal training in visual thinking strategy significantly improved the observational and descriptive skills of first- and second-year medical students and demonstrated that these skills were retained after six months, which implicates the direct usefulness of VTS in interpreting radiologic images. This is significant in that only a small amount of training led to significant changes in observational skills that were maintained after a six-month period. Our study is similar to a prior study by Agarwal et al. (2020), which used VTS training to increase the observational skills of medical students while analyzing EKG strips, abnormal chest radiographs, and clinical images of patients with physical findings. They demonstrated that the VTS training increased the total number of words used to describe the provided images, the time spent analyzing the images, as well as the number of clinically relevant observations made [6]. However, our study is unique in that a subjective element was introduced to grade the medical students on the quality and accuracy of descriptive observations. Additionally, it allowed for the assessment of abnormality recognition and subjective description of findings despite limited medical knowledge. It is interesting to note that there was a strong correlation between pre-test and post-test performance, which could imply different baseline characteristic observational skills in certain participants as well as participants who might benefit from further visual arts training. Further research would be needed to elucidate some of these underlying qualities.

To date, there have been few studies assessing the value of implementing a formal visual arts curriculum in the medical field and even fewer in the subspecialty of radiology. Most of these studies have attempted to blend visual literacy training into first-year medical students and nursing curricula, all of which have shown a significant improvement in the students’ observation skills of museum paintings unfamiliar to them in addition to patient photographs and various clinical scenarios [7-10]. In a study performed by Gurwin et al. (2018), the visual observation skills of medical students were assessed before and following a six-week visual literacy curriculum based on their abilities to describe retinal photographs, clinical photographs of faces involving ocular pathologies, and art images [11]. There was a statistically significant increase in their observational skills at the course’s end despite the lack of dedicated ophthalmology training, and the students reported utilizing the skills they learned during the visual literacy curriculum in their medical school practices. Visscher et al. (2019) had 50 third-year medical students analyze paintings depicting radiology encounters with patients using VTS and found that it challenged the negative stereotypes that medical students have of the radiology profession [12]. 

Early studies have also demonstrated the benefits of a formal visual arts curricula in various residencies including neurology to enhance communication and observational skills [13] and dermatology to increase the number of observations in medical images [14]. Finally, Goodman and Kelleher (2017) demonstrated a significant impact on radiology residents’ abilities to localize imaging abnormalities after a single session of visual arts training [15]. Despite the advantageous effects that these studies have demonstrated, few medical schools have implemented such a curriculum, and even fewer residencies.

A visual thinking strategy course or abbreviated introduction session would allow residencies to help hone observational skills in residents while taking a step back from the diagnostic aspects of radiology. Additionally, our approach included writing components to augment the VTS sessions, which afford practice of both oral and written specific and descriptive language, which is further suited to the field of radiology. Ultimately, being able to target observational intuition and develop this subset of subconscious observational skills would be highly advantageous in medical imaging.

Limitations

There were a few limitations to this study. The primary limitation was the virtual platform used for the VTS sessions with the art educators. The original study start date of May 2020 was postponed to September 2020 by the pandemic, preventing in-person sessions. While the virtual platform provided recordings, it also likely limited some discussions. There was also potential selection bias as students volunteered to participate, which likely attracted highly motivated medical students. It likely attracted medical students interested in radiology who were naturally highly visual observers.

Exposure to radiologic imaging was not limited to visual literacy training. Over the course of the six-month study, first-year medical students also had seven one-hour-long radiology lectures as a part of their previously established medical school curriculum including three hours of cardiothoracic radiology and four hours of abdominal imaging. Second-year medical students had three one-hour-long radiology lectures as a part of their previously established curriculum. While this counts as additional instruction during the course of the study, all students in the study had the same exposures.

The potential bias of test scorers is a limitation because the rubrics were previously established for each of the tests. While the scorers knew which test they were grading based on the rubric, objective grading criteria were considered that attempted to limit this bias.

The lack of a control group limits the interpretation of the results. However, the demonstration of a significant improvement for both first- and second-year medical students between the pre-test and the post-test does suggest associated improvement independent of concurrent medical training. There was also no long-term follow-up beyond six months to determine if the training had a lasting effect on radiologic observation skills. An additional limitation is the small sample size. The potential generalizability of VTS training to radiology trainees requires further investigation.

## Conclusions

A formal visual thinking strategy training curriculum showed significant improvement in observational and descriptive skills in first- and second-year medical students when describing radiologic images, which was retained after six months. This innovative form of teaching may facilitate active learning and improve reasoning and observational skills, particularly in subspecialties that utilize diagnostic imaging. Formal visual thinking strategy training or a formal visual arts curriculum could play a beneficial role in the visual detection and description of imaging in medical student and radiology training programs.

## Appendices

**Table 6:** Post-intervention survey questions The post-intervention survey was administered at the conclusion of the study following all data collection.

Post-Intervention Survey Questions
When you were participating in the visual thinking strategy course, did you notice any changes to how you approached a painting or a medical image?
If you felt that the course was beneficial, what was the most helpful aspect of the course?
Is there anything you wish that was covered that was omitted or that was covered more thoroughly?
What was the greatest hindrance of this study? (for example: time constraints, time commitment, confusing directions, etc.)
On a scale of 1-10 (1 being the least and 10 being the most), how likely are you to recommend this curriculum to other medical students?
On a scale of 1-10 (1 being the least and 10 being the most), how likely are you to use this curriculum in your future medical career?
Do you think that you will use this visual thinking strategy in the future? If so, how?
